# Editorial: Synthetic Live Biotherapeutic Products for Diseases

**DOI:** 10.3389/fmolb.2022.882743

**Published:** 2022-03-15

**Authors:** Ye Chen, Shuyi Zhang

**Affiliations:** ^1^ Institute of Synthetic Biology, Shenzhen Institutes of Advanced Technology, Chinese Academy of Sciences, Shenzhen, China; ^2^ School of Pharmaceutical Sciences, Tsinghua University, Beijing, China

**Keywords:** host-microbiota interaction, synthetic biology, part design, biotherapeutic drugs, protein engineering

Host-microbiota interactions have increasingly been shown to play important roles in supporting host health (Brown et al., 2019; Janney et al., 2020). In human, the gut microbiota contributes to several metabolic and immune-mediated diseases, such as obesity, intestinal inflammatory disease, malnutrition, and cancer. These broad host-microbiota interactions thus present unique opportunities to treat and cure diseases by modulating the structure and function of microbiota. By applying synthetic biology approaches, we can design non-pathogenic bacteria to sense and respond to environmental signals, and consume harmful compounds or deliver therapeutic effectors to cure diseases (Cubillos-Ruiz et al., 2021). However, due to the lack of fundamental understanding of host and microbiota interactions, and limited techniques to develop biological tools for manipulating microbial chassis derived from microbiota, there are still challenges in applications of these engineered biotherapeutics products. For this purpose, this research topic gathered articles that will promote our understanding of host-microbiota interactions and advance our ability to design synthetic live biotherapeutic products.

In order to modulate microbiota, we need to understand the composition of microbiota and their interactions with host. In this respect, Liu and coauthors used 16S rRNA gene amplicon and shallow metagenomic sequencing (SMS) to explore the gut microbiota directly from patient samples with colorectal disease (Liu et al.). They found that microbial diversities were similar between these two techniques, suggesting SMS as an economical approach for future clinical tests. This study identified dominant species, such as *Prevotella copri*, *Bacteroides dorei*, and *Bacteroides vulgatus*, that might be responsible for the progression of colorectal diseases. These strains may serve as chassis to diagnose, or even engineered to treat colorectal diseases. Next-generation sequencing (NGS) technology was also demonstrated to be a powerful technique in the study by Chen and coauthors to detect fetal copy number variant for non-invasive prenatal testing (Chen S. et al.). With increased read depth and reduced cost, it is foreseeable that NGS will have more contributions in the future.

Commensal bacteria produce a wide range of beneficial small molecules that can interact or even modulate host conditions, of which butyrate was found to exhibit extraordinary anti-cancer activities. Geng and coauthors demonstrated that in colorectal cancer cells, butyrate significantly inhibited glucose transport and glycolysis (Geng et al.). They discovered that abundance of membrane GLUT1 and cytoplasmic G6PD was greatly reduced and the GPR109a-AKT signaling pathway was vital in regulating these changes. More strikingly, they found that butyrate could promote chemotherapeutical efficacy of 5-fluorouracil on cancerous colonocytes, possibly due to exacerbated impairment of DNA synthesis. This study provides valuable information for the molecular mechanism of butyrate on glucose metabolism of colorectal cancer cells. These discoveries could promote the development of beneficial metabolites as therapeutical or adjuvant anti-cancer drugs. It is worth noting that butyrate could improve the antivirus capacity of human (Trompette et al., 2018), which makes it a special chemical worth further investigating especially in the COVID-19 pandemic.

Rather than relying on existing microbiota and molecular parts, with the advances in synthetic biology, we can design and engineer valuable regulatory parts as needed. Li and coauthors employed molecular dynamics simulation and the Molecular Mechanics Generalized Born Surface Area (MM-GB/SA) method to artificially designed transcription factors with different binding affinities (Li et al.). They focused on LuxR, a bacterial quorum sensing regulator from *Vibrio fischeri*, which can be engineered to sense environmental signals and control overall microbiota behaviors. In this work, they identified a key residue, isoleucine-46, when modified to F or R, can have different binding affinity to the same substrate 3-oxo-hexanoyl-homoserine lactone. They implemented the designed transcription factors into genetic circuits in *E. coli* to detect target bacterial pathogens such as *Yersinia pestis*. The overall workflow showcased how synthetic biology, especially protein engineering and circuit design, can facilitate microbiota engineering for bio-therapeutical applications.

Several excellent reviews were also assembled for our readers in this topic. Zhang and coauthors provided a comprehensive review of the recent advances in understanding mechanisms of microbiomes as well as synthetic biology technologies that can be utilized to prevent, detect, and treat various diseases (Zhang et al.). Chen and coauthors summarized impacts of the host-microbiome interactions on osteomyelitis pathogenesis, covering direct and indirect microbiome-associated osteomyelitis (Chen J. et al.). Finally, Yang and coauthors reviewed the immune responses of severe COVID-19 in the gastrointestinal microenvironment and the gut-lung axis (Yang et al.). This review highlighted the potential of using probiotics as an alternative drug to alleviate or even prevent severe COVID-19 outcomes.

We hope the articles and reviews collected in this research topic can show how fascinating this emerging field in the intersection of synthetic biology and microbiota is, and the great therapeutical potentials this field might offer. We want to take this chance to thank all the authors and reviewers for contributing to this special topic issue. As we are starting Volume II, we encourage and welcome more experts from this field to submit their research work to our topic.
